# Risk factors for cardiovascular disease among people with mental illness in Namibia

**DOI:** 10.4314/gmj.v56i4.7

**Published:** 2022-12

**Authors:** Ndahambelela F N Mthoko, Lilian Pazvakawambwa, Marja Leonhardt, Lars Lien

**Affiliations:** 1 Mental Health Care Centre, Private Bag 13215, Windhoek, Namibia; 2 Department of Statistics and Population Studies, University of Namibia, P. O. Box 40933, Ausspannplatz, Windhoek, Namibia; 3 Norwegian National Advisory Unit on Concurrent Substance Abuse and Mental Health Disorders, Innlandet Hospital Trust, Post box 104, 2381 Brumunddal, Norway; 4 Faculty of Health Studies, VID – Specialized University, Oslo, Norway; 5 Department of Health and Social Science, Innlandet University of Applied Science, Elverum, Norway

**Keywords:** Mental illness, cardiovascular disease, risk factor, Namibia, prevalence

## Abstract

**Objectives:**

To determine the prevalence of risk factors for cardiovascular disease (CVD) among people with mental illness attending the Mental Health Care Centre, Windhoek, Namibia

**Design:**

Observational, cross-sectional study.

**Setting:**

Mental health Care Centre, Windhoek Central Hospital. Namibia

**Participants:**

Adult patients with a mental illness attending the Mental Health Care Centre, Windhoek.

**Data collection:**

Within a systematic random sampling method, 385 adult patients with mental illness were recruited between May and December 2017.

**Statistical analysis:**

Validated assessment tools were used. Descriptive summary statistics and Chi-squared tests of association were conducted.

**Results:**

One-third (31.7%) of participants used alcohol, 21% used nicotine, 21.3% had hypertension, 55% were over-weight or obese, 59.2% of females and 11.5% of males had abdominal obesity. About twenty per cent (19.9%) of participants did meet the World Health Organisation recommended level of activity, while more than two-thirds of participants did not participate in moderate or vigorous physical activities. The patient's psychiatric condition was significantly associated with alcohol use (Chi-square=20.450, p=0.002) and physical activity (Chi-square=20.989, p=0.002). The psychiatric condition was not associated with the waist circumference and gender of the participant.

**Conclusions:**

The increased prevalence of CVD risk factors in people with mental illness calls for mental health practitioners to screen, monitor and manage these risk factors regularly. Systematically screening and monitoring for cardiovascular risk factors is likely to contribute to National targets and significantly impact cardiovascular morbidity and mortality in people with mental illness.

**Funding:**

This work was financed by internal resources of the Mental Health Care Centre, Windhoek Central Hospital

## Introduction

Mental illness is associated with an increased risk of premature death.^1^ Persons with mental illness die approximately 15-20 years earlier than the general population^2^ Most of the deaths among persons with mental illness are not due to their mental but due to physical illness^3^ Cardiovascular disease (CVD) represents the most common natural cause of excess mortality.^4^ Globally, CVD are a leading cause of death. More people die annually from CVD than from any other cause. In 2019, there were an estimated 17.9 million deaths due to CVD, representing 33% of all global deaths, and over three-quarters of CVD deaths occur in low- and middle-income countries.^5^ In 2015, about 1.2 million deaths in sub-Saharan Africa (SSA) were attributable to CVD.^6^ According to the World Health Organization (WHO) Non-communicable diseases (NCDs) Country Profiles for 2014, CVD are the most common NCDs and the third most common cause of death in Namibia, next to HIV/AIDS and Tuberculosis, accounting for 21% of all mortality.) The CVD burden of SSA, projected to double by 2030, is predominantly driven by increased rates of hypertension, smoking, and obesity.

A study by Owolabi et al. found that hypertension, unhealthy diet, physical inactivity, and current cigarette smoking were independently associated with stroke occurrence.^8^ The risk factors for CVD in Namibia include hypertension, smoking, lack of physical exercise, harmful use of alcohol, unhealthy diets and obesity.^6^

Hypertension is a major cause of CVD in Africa.^9^, ^10^ Studies reported a prevalence range of 15–70% for hypertension for the period from 1999 to 2013 in sub-Saharan African countries.^11^ In 2018, the prevalence of hypertension in Namibia was 22%.^7^ Evidence showed that people with mental illness, compared with the general population, have two-three times the risk of hypertension.^12^

Regular physical activity reduces the risk of coronary heart disease, stroke, diabetes, hypertension, and depression.^13^ People experiencing mental illness are less likely to engage in physical activity than the general population.^14^ Mental health problems may reduce an individual's motivation to get out, and exercise and problems with social interactions with others may predispose the individual to stay at home.^15^ In many African countries, an increase in weight profile among inhabitants, has been directly implicated in rising cardiovascular risk levels from adolescence onwards.^16^

Most CVD can be prevented by addressing risk factors. Thus, Namibia aims for a 15% relative reduction in the prevalence of raised blood pressure and tobacco use and a 7% relative reduction in the use of alcohol and the prevalence of insufficient physical activity by 2022.^17^ To achieve these targets, health practitioners have to identify and manage these risk factors among all patients including persons with mental illness. To our knowledge, estimates of risk factors for CVD among the mentally ill in Namibia are currently lacking. Therefore, the objective of this study was to determine the prevalence of risk factors for CVD among the mentally ill attending the Mental Health Care Centre, Windhoek (MHCCW).

## Methods

### Study design, setting, and participants

This observational, cross-sectional study is part of a larger project studying Namibia's epidemiology of persons with mental illness. The results of a sub-study examining substance use among this group have been published elsewhere.^18^ The present study was conducted between May and December 2017 at the MHCCW, a referral and teaching health facility. The study population was service users with mental illness attending the outpatient service of the MHCCW. Inclusion criteria were adult patients aged 18 years and above and those who could independently provide informed consent to participate in the study. Exclusion criteria were patients below 18 years and persons who could not give informed consent.

### Data collection

**Figure 1:**
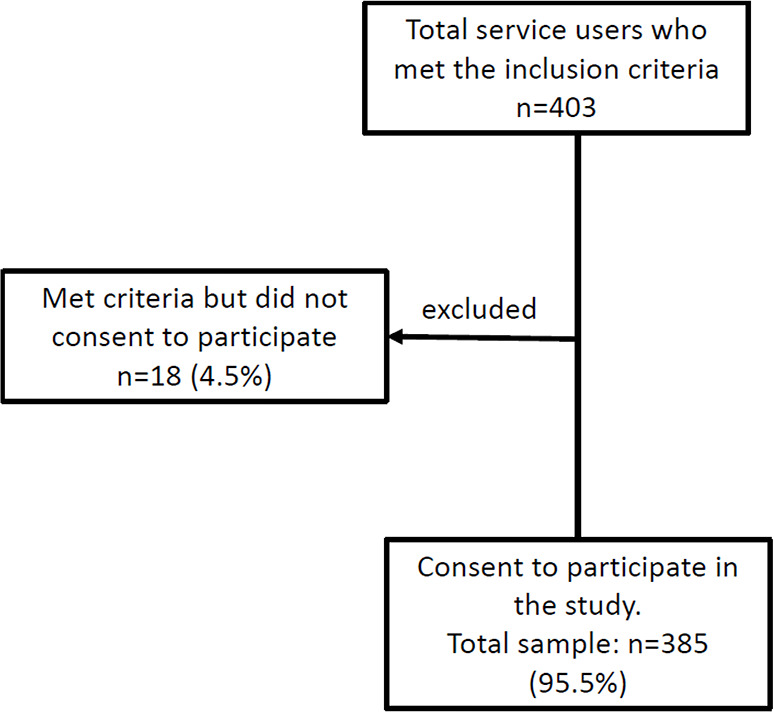
Flow chart of the inclusion process

A systematic random sampling method was used. Every fourth patient was given a number to take part in the study. In cases where the fourth patient was a child or could not consent, the subsequent patient was assessed. Medical officers at the MHCCW referred patients to the researchers after routine assessment and management. Patients were sorted out for inclusion criteria; those who did not meet the criteria were thanked and excluded from the study. Explanations about the study were made to eligible and willing participants, and an informed consent form was signed. Participants were assured that the participation was completely voluntary, there would be no material gain from the study, they could withdraw at any time during the interview, and that refusal to participate would not affect their health services/benefits entitlement. Consented participants were then interviewed, and the researchers measured their weight, height, waist circumference (WC), and blood pressure (BP). The data inclusion process is shown in Figure 1.

### Measures

Study variables included gender, age at enrollment, employment, marital, and socio-economic status (low, medium, or high); where high socio-economic status was defined as having held a white collar/managerial job; medium socio-economic status was defined as other types of employment and low defined as unemployment. Information about psychiatric and medical diagnoses was obtained from interviewing the participants and reviewing the participant's medical records. The Alcohol Use Disorders Identification Test (AUDIT)^19^, and the Fagerström Test for Nicotine Dependence^20^ were administered to all participants.

Information about BP was obtained by reviewing patients' medical records and measuring their BP. Patients were seated to determine systolic and diastolic BP (SBP and DBP; mm Hg). BP was measured with a standard mercury sphygmomanometer after the participant sat and rested for 3-5 minutes. Two BP measurements were taken for each participant with an interval of 2-3 minutes between the readings, averaged across two readings and classified into normal (129-139/80–89 mmHg), grade 1 (140-159/90-99 mmHg), grade 2 (160-179/100-109mmHg), and grade 3 (≥180/≥110mmHg).^21^

Body mass index (BMI) was calculated as the weight in kilograms divided by height in meters squared and categorized as normal weight (BMI<25 kg/m^2^), overweight (BMI 25.0–29.9 kg/m^2^), or obese (BMI≥30 kg/m^2^).^22^ Waist Circumference (WC) was used to classify participants as having a normal abdominal circumference (WC < 80 cm for women and 94 cm for men), a moderate (WC between 80 and 88 cm for women or 94 and 102 cm for men) or a severe (WC > 88/102 cm for women and men, respectively). Abdominal obesity was defined as waist circumference >102 cm for men and >88 cm for women.^23^, ^24^

The WHO STEPS (STEPwise approach to surveillance) protocol to measure the WC was followed. It was measured exactly between the lower rib and the upper edge of the hip bone by using a flexible measuring tape.^24^ All measurements were performed by two health professionals to ensure measurement validity.

### Data analysis and processing

The data were analyzed using IBM SPSS Statistics for Windows, Version 23.0. Armonk, NY. The dataset was coded before data entry and assessed for errors and outliers. Descriptive summary statistics in the form of centrality, dispersion and frequency distributions and tables were used to profile sociodemographic characteristics; psychiatric conditions; alcohol and tobacco use, BP, BMI and WC.

Cross tabulations and Chi-squared tests of association were conducted to establish whether there were significant associations between psychiatric conditions and hypertension, psychiatric conditions and waist circumference, psychiatric condition and physical activity, and BMI and physical activity. In all analyses, a probability value of p< 0.05 was considered statistically significant.

### Ethics

Approval to carry out the study was obtained from the Ethics and Research Committee, Ministry of Health and Social Services, Windhoek, Namibia, with reference no 17/3/3. Authorization of patients' participation was also sought from the institution's head. All participants gave written consent to participate after the study procedures had been explained to them. The patients who did not consent to participate were excluded. To secure confidentiality, serial numbers were assigned instead of names.

## Results

### Characteristics of the study population

The sociodemographic and clinical characteristics and use of alcohol and tobacco by the participants are presented in Table 1. Of the 385 participants, 52.2% were males, and 47.8% were females. The mean age at enrolment was 37.14 ± 10.33years. Participants were predominantly single (79.0%), with secondary education (67.8%). About half of the participants (51.4%) were unemployed, and sixty per cent were of low socio-economic status. Regarding psychiatric diagnoses, 54.5% had schizophrenia spectrum disorders, 22.3% had depressive disorders, and 17.7% had bipolar and related disorders.

**Table 1:** Association of demographic and clinic Characteristics of participants with the use of alcohol and tobacco

Sociodemographic and clinical	Entire sample	Alcohol use	Tobacco use
**Sex**			
**Female**	184(47.8%)	36 (19.6%)	18(9.8%)
**Male**	201(52.2%)	86(42.7%)	62 (30.8%)
**P-value**		<0.001*	<0001*
**Age group in years**			
**Youth (18–35)**	180(46.8%)	67(37.2%)	35(19.4%)
**Middle age (36–55)**	185(48.0%)	53(28.6%)	38 (20.5%)
**Older people (56 and above)**	20(5.2%)	2(10.0%)	7 (35.0%)
**P-value**		0.012*	0.266
**Marital Status**			
**Single**	304(79%)	100 (32.9%)	84(27.6%)
**Married**	58(15.1%)	16(27.6%)	10 (17.2%)
**Separated**	13(3.4%)	6 (46.1%)	4(30.8%)
**Widowed**	10(2.6%)	0 (0.0%)	2(20.0%)
**P-value**		0.094	0.739
**Education**			
**None**	7(1.8%)	5(71.4%)	3 (42.8%)
**Primary**	58(15.1%)	15 (25.9%)	12(20.7%)
**Secondary**	261(67.8%)	80 (30.6%)	57 (21.8%)
**Tertiary**	56(14.5%)	22(39.3%)	11(19.6%)
**P-value**		0.415	0.574
**Occupation**			
**Student**	28(7.3%)	9 (32.1%)	1(3.6%)
**Self-employed**	25(6.5%)	9 (36.0%)	8 (32.0%)
**Employed**	117(30.4%)	42 (35.9%)	17 (14.5%)
**Unemployed**	198(51.4%)	61(30.8%)	50 (26.5%)
**Other**	16(4.2%)	1(6.2%)	4(25.0%)
**P-value**		0.070	0.011*
**Socio-economic status**			
**Low (Never employed)**	230(59.7%)	69 (30.0%)	55 (23.9%)
**Medium (other employment)**	127(33.0%)	43 (33.8%)	21(16.5%)
**High (White collar job)**	22(5.7%)	10 (45.4%)	4(18.1%)
**P-value**		0.641	0.186
**Psychiatric conditions**			
**Schizophrenia spectrum and other psychotic** **disorders**	210(54.5%)	68 (32.4%)	45 (21.4%)
**Bipolar and related disorders**	68(17.7%)	12 (17.6%)	13(19.1%)
**Depressive disorder**	86(22.3%)	36 (43.9%)	18 (21.9%)
**Others**	21(5.5%)	4(19.0%)	2(9.5%)
**P-value**		0.002*	0.073

* p-value < 0.05

^1^Includes other forms of occupation, which could not be nearer assessed.

^2^Includes other forms of psychiatric conditions which are not nearer specified.

Out of the participants, 122 (31.7%) used alcohol, and 80 (20.8%) smoked tobacco. Alcohol use was common among males 42.7%, youth 37.2%, and those with depressive disorders 43.9%. About 24% of the participants used alcohol harmfully/hazardously or were possibly dependent on alcohol. There was a significant association between the patient's psychiatric condition and alcohol use (Chi-square=20.450, p=0.002).

### Blood pressure

Measured BPs were compared to the patient's existing records to confirm the potential elevation of BP. A BP of 140/90mmHg or greater indicated hypertension. We diagnosed 81(21.3%) participants with hypertension, with a BP of 140/90mmHg or greater. Among the participants, 14.8% had grade 1, 5.2% had grade 2, and 1.3% had grade 3 hypertension. Table 2 shows the association between psychiatric conditions and the grades of hypertension. Twenty-six per cent of the participants with a diagnosis of schizophrenia spectrum disorder, 16.3% of those with depressive disorders, and 14.7% of those with bipolar and related disorders had hypertension.

**Table 2:** Association between psychiatric conditions and the grades of hypertension

Psychiatric Condition	Entire sample	Grades of hypertension				

		Normal BP (129–139/80–89 mmHg)	Grade 1 Hypertension (140–159/ 90–99 mmHg)	Grade 2 Hypertension (160–179/100–109 mmHg)	Grade 3 Hypertension (≥180/≥110 mmHg)	
**Schizophrenia spectrum and** **other psychotic disorders**	210(54.5%)	155(73,8%)	42 (20.0%)	10 (4.8%)	3(1.4%)	Chi-square=31.405, p=0.026
**Bipolar and related disorders**	68 (17.7%)	58 (85.3%)	3(4.4%)	5(7.3%)	2(3.0%)	
**Depressive disorder**	86 (22.3%)	72 (83.7%)	10(11.6%)	4(4.7%)	0	
**Others** *	21(5.5%)	18 (85.7%)	2(9.5%)	1(4.8%)	0	
**Total**	385	303(78.7%)	57(14.8%)	20(5.2%)	5(1.3%)	

* Includes other forms of psychiatric conditions, which are not nearer specified.

### BMI and Waist circumference

The participants' mean weight was 71.98 ± 15.66kg, mean height was 165.96 ± 9.28cm and mean BMI of 25.95kg/m2±6.73kg/m2. Ninety (23.4%) participants had a BMI over 30, indicating obesity. Another 122 (31.7%) participants had a BMI between 26 and 29, indicating they were overweight. In combination, 55% of the participants with mental disorders were overweight or obese. Regarding psychiatric conditions, 18.1% of the participants with schizophrenia spectrum and other psychotic disorders, 38.2% of those with bipolar and related disorders and 19.7% of those with depressive disorders were obese.

The mean WC of the participants was 88.09cm±13.66cm. Of the female participants, 109 (59.2%) had a waist circumference indicating a high risk of disease or abdominal obesity, while the males were 23 (11.5%). Fifty-seven per cent of participants with depressive disorder, 73.5% of participants with Bipolar and related disorder and 46.2% of participants with schizophrenia spectrum and other psychotic disorder had a waist circumference indicating either increased risk or high risk of disease.

The association of psychiatric conditions with waist circumference is presented in Table 3. There were no significant associations between the waist circumference and psychiatric conditions for male (Chi-square=12.416, p=0.053) and female (Chi-square=18.868, p=0.092) participants.

**Table 3:** The association of psychiatric conditions with WC

Psychiatric conditions	Entire sample		WC of the participants						

			Low risk of disease (<80cm for women;< 94 cm for men)		Increased risk of disease (80–87 for women; 94–101cm for men)		High risk of disease (>88cm for women>104 cm for men)		Chi square P-value
	Female	Male	Female	Male	Female	Male	Female	Male	
**Schizophrenia spectrum and** **other psychotic disorders (n** **=210)**	68 (37.0%)	142 (70.6%)	10 (14.7%)	103 (72.5%)	9 (13.2%)	23(16.2%)	49(72.0%)	16(11.3%)	Females: p=0.053 Males p=0.092
**Bipolar and related disorders** **(n =68)**	45(24.4%)	23(11.4%)	8 (17.8%)	10(43.5%)	7 (15.5%)	9(39.1%)	30(66.7%)	4 (17.4%)	
**Depressive disorder (n =86)**	52(28.3%)	34 (17.0%)	12(23.0%)	25(73.5%)	13(25.0%)	6 (17.6%)	27 (51.9%)	3(8.8%)	
Others* (n = 21)	19(10.3%)	2(1.0%)	10(52.6%)	1(50.0%)	6(31.6%)	1(50.0%)	3(15.8%)	0(0.0%)	
**Total**	184(47.8%)	201(52,2%)	40(21.7%)	139(69.1%)	35(19.0%)	39(19.4%)	109(59.2%)	23(11.5%)	

* Includes other forms of psychiatric conditions which are not nearer specified.

### Physical activity and BMI

About twenty per cent (19.9%) of participants did meet the WHO recommended level of activity of 150 minutes of moderate-intensity physical activity throughout the week or 75 minutes of vigorous-intensity physical activity throughout the week.

Among the participants, 76.6% and 71.0% did not participate in moderate and vigorous activity, respectively.

There was a significant association between BMI and vigorous physical activity (Chi-square=19.606, p=0.003).

Among the participants who were obese, 90% did not do vigorous physical activities. The association between psychiatric condition, BMI and the level of physical inactivity is presented in Table 4. There was a significant association between psychiatric conditions and physical activity (Chi-square=20.989, p=0.002).

**Table 4:** Association between psychiatric condition, BMI, and physical inactivity

Physical conditions	Light activity (Chi Square=6.733, p=0.665) N (%)					Moderate activity (Chi Square=19.034, p=0.025) N (%)					Vigorous activity (Chi Square= 17.905, p=0.036) N (%)				
	
	None	1 hour per week	1 hour 3 times per week	1 hour daily	Total	None	1hour per week	1 hour 3 times per week	1 hour daily	Total	None	1hour per week	1 hour 3 times per week	1 hour daily	Total
Schizophrenia spectrum and other psychotic disorders	1 (0.5)	3 (1.4)	0 (0.0)	206 (98.1)	210 (100)	165 (82.9)	10 (13.6)	9 (4.5)	15 (7.5)	199 (100)	155 (77.1)	26 (12.9)	9 (4.5)	11 (5.5)	201 (100)
Bipolar and related disorders	0 (0)	0 (0)	1 (1.5)	67 (98.5)	68 (100)	48 (72.7)	9 (13.6)	2 (3.0)	7 (10.6)	66 (100)	54 (80.6)	9 (13.4)	0 (0.0)	4 (6.0)	67 (100)
Depressive disorder	0 (0)	1 (1.3)	0 (0)	79 (98.8)	80 (100)	66 (86.8)	2 (2.6)	1 (1.3)	7 (9.2)	76 (100)	60 (76.9)	4 (5.1)	5 (6.4)	9 (11.5)	78 (100)
Others*	0 (0)	0 (0)	0 (0)	24 (100)	24 (100)	16 (66.7)	2 (2.6)	0 (0)	6 (25)	24 (100)	24 (100)	0 (0)	0 (0)	0 (0)	24 (100)
**BMI of participants**	**Light activity** (Chi Square=23.557, p=0.005) N (%)					**Moderate activity** (Chi Square =21.912, p=0.009) N (%)					**Vigorous activity** (Chi Square =35.739, p≤0.001) N (%)				
	**None**	**1 hour** **per** **week**	**1 hour 3** **times per** **week**	**1** **hour** **daily**	**Total**	**None**	**1hour** **per** **week**	**1 hour** **3 times** **per** **week**	**1** **hour** **daily**	**Total**	**None**	**1hour** **per** **week**	**1 hour** **3 times** **per** **week**	**1** **hour** **daily**	**Total**
Malnourished BMI <18.4kg/m^2^	0 (0)	2 (11.1)	0 (0.0)	16 (88.9)	18 (100)	12 (66.7)	0 (0)	2 (11.1)	4 (22.2)	18 (100)	10 (62.5)	6 (37.5)	0 (0)	0 (0)	16 (100)
Healthy weight (BMI =18.5 -24.9 kg/m^2^	1 (0.6)	0 (0)	1 (0.6)	152 (98.7)	154 (100)	122 (82.4)	9 (6.1)	7 (4.7)	10 (6.8)	148 (100)	121 (81.2)	17 (11.4)	6 (4.0)	5 (3.4)	149 (100)
Overweight (BMI =25.0 - 29.0kg/m^2^	0 (0)	2 (1.7)	0 (0)	118 (98.3)	120 (100)	85 (76.6)	7 (6.3)	1 (0.9)	18 (16.2)	111 (100)	81 (69.8)	12 (10.3)	7 (6.0)	16 (13.8)	116 (100)
Obese BMI ≥ 30kg/m^2^	0 (0)	0 (0)	0 (0)	90 (100)	90 (100)	76 (86.4)	7 (8)	2 (2.3)	3 (3.4)	88 (100)	81 (91)	4 (4.5)	1 (1.1)	3 (3.4)	89 (100)

* Includes other forms of psychiatric conditions, which are not nearer specified.

More than two-thirds of participants with schizophrenia spectrum and other psychotic disorders (73.8%), major depressive disorders (69.8%), and bipolar and related disorders (79.4% did not participate in vigorous physical activities.

## Discussion

The study aimed to identify the prevalence of risk factors for CVD among the service users at the MHCCW. The results of the study show an increased prevalence of risk factors for CVD in patients with mental illness especially related to overweight and obesity.

The highest discrepancy between patients attending the MHCCW and the general population was for those who did not meet the WHO-recommended level of activity, as up to two-thirds of participants did not participate in moderate or vigorous physical activities.

There were some notable differences in risk profiles among the three different diseases. Those with psychosis and schizophrenia spectrum diseases had a higher grade 1 hypertension, and patients with bipolar-related disorders had the highest alcohol consumption.

For all other risk factors, there were only minor non-significant differences. Considering recent studies on CVD morbidity and mortality showing that people with schizophrenia are at particularly high risk compared to people with other mental disorders^25^, the finding might be attributed to attenuation of the schizophrenia group with people having less severe and more limited psychosis.

In earlier studies, the most important risk factor for developing CVD among people with mental health disorders has been related to smoking, alcohol abuse and the use of illegal substances.^26^, ^27^ Unfortunately, we did not measure the use of substances other than nicotine and alcohol. Alcohol and tobacco are, however, major risk factors for CVD.^28^ More people in the study consumed alcohol compared to 27.7% of Namibians aged 15 years and above who reported consuming alcohol.^7^ Most people who used alcohol were likely to use tobacco. The prevalence of smoking among the participants (21%) was slightly higher than the general population (20%).^7^

The low rate of smoking and alcohol use differs from studies on the same population in high-income countries. Many patients with psychosis misuse alcohol.^29^ Further, several studies indicate that more than 70% of patients admitted to mental health or addiction units are current smokers.^30^, ^31^ These variations also reveal differences in CVD morbidity and mortality that might be lower in Namibia compared to high-income countries. There are few studies on smoking and alcohol use rates in mental hospitals in African countries, but the population level figures are comparable.^32^,^33^

Hypertension has been identified as the leading cause of mortality and the third cause of disability-adjusted life years worldwide^6^, and a major and preventable risk factor for CVD in Namibia.^17^ The prevalence of hypertension in this study (21.3%) is almost equal to the general population in Namibia (22%).^7^ The reason might be that the most important risk factors for hypertension, like too much salt, fat and sugar in the diet and lack of fruit and vegetables, are the same in both populations.^34^, ^35^

A systematic review by Pan and colleagues showed an association between mental illness and hypertension.^36^ In the present study, one-fourth of the participants had raised BP, 26.2% of those with a schizophrenia spectrum disorder, 16.3% of those with depressive disorders, and 14.7% of those with bipolar and related disorders had hypertension. Many patients with mental illness have experienced neglect or abuse during childhood.^37^ These experiences tend to lead to a higher level of stress reactivity in the body with higher HPA axis output and which certainly also increases the risk for hypertension.^38^

Cross-sectional studies of younger adults from African countries have reported that less-than-optimal activity levels are linked to increased BMI and BP.^39^,^40^

The health benefits of physical activity include reduced risks for premature chronic health conditions, prevention of unhealthy weight gain, and reduced mortality and extended life expectancy.^41^ Nyboe and Lund reported that despite the well-established health benefits of physically-active lifestyles, people experiencing mental illness are less likely to engage in physical activity than the general population.^14^

Results show that only 19.9% of the participants met the WHO-recommended level of physical activity. Research shows that people with mental illness engage in significantly less vigorous activity than healthy controls.^42^ Seventy-six percent of the participants in this study did not engage in vigorous physical activity.

Other studies demonstrate that less than half of the people with schizophrenia^43^, bipolar disorder^44^ and major depression 45 meet the recommended physical activity levels of 150 minutes of moderate-vigorous physical activity per week. In our study, more than two-thirds of participants with schizophrenia spectrum and other psychotic disorders, major depressive disorders and bipolar and related disorders did not participate in moderate or vigorous physical activity. Factors contributing to low physical activity levels of people with mental illness include medication-related weight gain, sedation, and lowered self-esteem.^46^, ^47^

Physical inactivity is also predictive of various adverse health outcomes, including obesity, diabetes and medical co-morbidity among people with mental illness.^7^ Obesity is reported to be a risk factor for multiple chronic diseases and conditions, including hypertension, hypercholesterolemia and coronary heart disease.^48^ Obesity is also associated with an increased risk for several mental disorders, such as depression and bipolar disorder, which are associated with great disease burdens and increased mortality, disability, and reduced quality of life.^32^, ^49^ People with mental illness have a significantly higher risk of obesity than those without mental illness.^50^ The prevalence of obesity, defined as a BMI of ≥ 30 kg/m^2^, was 23.4%. Over half of the participants were either overweight or obese. Obesity among the adults was 15% in Namibia^7^, less than the 23.4% found among the participants in this study.

Results indicated significant relationships between psychiatric conditions and BMI. Bipolar and related disorders were found to be associated with obesity. Abdominal obesity, defined as WC of > 102 cm for men and > 88 cm for women, was 59.2% for females and 12.4% for men. Although other research shows associations between mental illness and WC48-50, there were no significant associations in our study. Research attributed high rates of obesity among individuals with mental illness to poor diet, medication and sedentary lifestyle.^51^

Some scholars claim that the higher mortality and morbidity of CVD among people with mental illness are merely a reflection of disadvantaged socio-economic status.^52^ From this study, we found that only 15% were married, 14% had tertiary education and more than 50 % were unemployed. From earlier studies, we know that poverty is a vicious cycle where mental disorders lead to poverty and that poverty increase the risk of mental health problems.^53^ Thus, the low socio-economic status may impair physical activity and the ability to buy healthy food.

This study has several limitations. First, it was a cross-sectional design; therefore, the inability to rule out other unknown confounders could have influenced the findings related to obesity (e.g. dietary habits, medication). Second, participation in physical activity was based on self-report by participants. Therefore, the results could be affected by response bias. Third, the study did not cover all cardiovascular risk factors, leaving blood glucose, blood lipids, diet composition, and genetic factors. Despite limitations, the findings draw attention to the need for healthcare practitioners to closely screen and monitor the physical health of people with mental illness. Efforts to reduce the excess burden of morbidity and mortality among people with mental illness need to address the problem of CVD risk factors.

## Conclusion

The findings of this study provide a meaningful contribution to the limited research on CVD risk factors in persons with severe mental illness in Namibia. The increased rates of CVD risk factors among this target group call for mental health practitioners to screen, monitor and manage these risk factors regularly. Systematically screening and monitoring for cardiovascular risk factors is likely to contribute to the Namibian “National targets” and to have a significant impact on cardiovascular morbidity and mortality in people with mental illness in Namibia
